# GCN2 enhances host survival and drives eIF2α phosphorylation during mouse adenovirus type 1 infection

**DOI:** 10.1128/jvi.01288-25

**Published:** 2025-09-24

**Authors:** Luiza A. Castro Jorge, Daniel F. Edwards, Rosario Labastida, Danielle E. Goodman, Estela A. Pereira, Oded Foreman, Katherine R. Spindler

**Affiliations:** 1 Department of Microbiology and Immunology, University of Michigan 1259, Ann Arbor, Michigan, USA; 2 Department of Pathology, Genentech Inc. 7412 , South San Francisco, California, USA; Tufts University School of Medicine, Boston, Massachusetts, USA

**Keywords:** integrated stress response, PKR, protein kinase R, general control nonderepressible 2

## Abstract

The integrated stress response (ISR) is a cellular signaling pathway that reduces protein synthesis in the face of cellular stress, including viral infection. Two eukaryotic initiation factor 2α (eIF2α) kinases, protein kinase R (PKR) and general control nonderepressible 2 (GCN2), are commonly activated during viral infections. Mouse adenovirus type 1 (MAV-1) infection leads to a steep reduction of PKR levels by proteasomal degradation. We assayed whether GCN2, a sensor of amino acid starvation and UV damage, plays a role in the ISR to MAV-1 infection. There was more phosphorylated GCN2 in MAV-1-infected cells, and its activation was dependent on virus replication since UV-inactivated virus was not able to increase the phosphorylation of GCN2. Infected *Eif2ak4^tm1.2Dron^* mice (designated here *Gcn2^−/^*^−^ mice) had lower survival than wild-type (WT) mice, but results indicated that this was not due to increased viral replication. Both *Gcn2^−/^*^−^ and WT mice developed multifocal brain parenchymal microhemorrhages during infection. While *Gcn2^−/^*^−^ animals had more lesions, their higher mortality is likely not due to the microhemorrhages alone. Cytokine RNA and protein assays of WT and *Gcn2^−/^*^−^ mice only showed a difference for IL- β levels, which were higher in *Gcn2^−/^*^−^ mice. Our results also indicate that of the two eIF2α kinases, PKR and GCN2, GCN2 is the primary inducer of phosphorylated-eIF2α during MAV-1 infection. GCN2 is thus antiviral and contributes to the host response to MAV-1 infection.

**IMPORTANCE:**

Cells often respond to viral infection by activation of the host protein kinase R (PKR), part of the integrated stress response (ISR). We show that a second host protein kinase, general control nonderepressible 2 (GCN2), is activated by phosphorylation in response to mouse adenovirus type 1 (MAV-1) infection. Our results indicate GCN2 is antiviral: without it, the mortality in MAV-1-infected mouse is higher. Furthermore, the data show that GCN2, rather than PKR, is the main inducer of eIf2α phosphorylation (and thus the ISR) upon MAV-1 infection. This is consistent with PKR exerting antiviral effects in MAV-1 infections through a pathway independent of eIf2α phosphorylation.

## INTRODUCTION

Virus infection relies on the host translation machinery to produce viral proteins necessary for virus replication. Therefore, the reduction of protein synthesis is an effective mechanism employed by the host to resist viral infection. Host cells have several ways to reduce protein synthesis. One involves the integrated stress response (ISR), which is an intricate signaling pathway that can be induced by four different eIF2α kinases: heme-regulated inhibitor of translation (HRI and EIF2AK1), protein kinase R (PKR and EIF2AK2), PKR-like endoplasmic reticulum kinase (PEK/PERK and EIF2AK3), and general control nonderepressible 2 (GCN2 and EIF2AK4) (1, 2). Each of these eIF2α-kinases responds to distinct environmental stresses. HRI monitors changes in hemoglobin levels (3); PKR senses dsRNA during viral infection (4); PERK is activated by endoplasmic reticulum (ER) stress (5); and GCN2 detects amino acid starvation, UV damage, and viral infection (6, 7). However, they all converge on eIF2α phosphorylation, which broadly reduces translation but allows selective translation of genes with upstream ORFs in their 5′ untranslated region (8).

Many viruses have mechanisms that counteract the action of eIF2α kinases, and PKR has been one of the most studied eIF2α kinases in this context. Some viruses block PKR activation by encoding proteins that sequester dsRNA, blocking its interaction with PKR, such as influenza virus NS1 (9), vaccinia virus E3L (10–12), and Ebola virus VP35 (13). Other viruses encode proteins or RNAs that bind directly to PKR, inhibiting its activation, such as HIV-1 Tat protein (14), herpes simplex virus US11 (15, 16), and human adenovirus (HAdV) VA RNAs (17). Another mechanism is PKR degradation, employed by poliovirus (18), Rift Valley fever virus (19, 20), Toscana virus (21), foot-and-mouth-disease virus (22), and enterovirus A71 (23). We reported that mouse adenovirus type 1 (MAV-1; also known as MAdV-1) can also degrade PKR, being the first DNA virus identified that does so (24, 25). Recently, fowl adenovirus was identified to also degrade PKR (26). Together, these results indicate that ISR signaling is important during adenovirus infection.

MAV-1 is in the *Adenoviridae* family, and it causes both acute and persistent infection in mice, leading to a dose-dependent encephalitis in susceptible mice (27, 28). MAV-1 enables the investigation of adenovirus pathogenesis in a small animal host; the study of HAdV pathogenesis in an animal model is difficult because adenoviruses are species-specific (29). However, MAV-1 pathogenicity and tropism differ from known HAdVs: MAV-1 infects endothelial cells and monocytes, causing encephalitis and myocarditis, while HAdVs infect epithelial cells, leading to upper respiratory and GI tract infections and conjunctivitis (27, 30, 31). We and others have been investigating innate and adaptive immune responses to MAV-1. The ISR likely plays an important role in MAV-1 pathogenesis not only through PKR (24, 25, 32) but also through GCN2. A loss-of-function mutation in *Eif2ak4*, encoding GCN2, the *Atchoum* (*Atc*) mutation, led to an increased susceptibility to infection in peritoneal macrophages by human adenovirus (33).

GCN2’s role in viral infections is less well appreciated or understood than that of PKR. However, several studies have shown that GCN2 also inhibits viral infections (34). Sindbis virus infection of mouse embryonic fibroblasts (MEFs) activates GCN2, and infection of *Gcn2^−/^*^−^ mice results in more viral replication than in control mice (35). Similarly, *Atc* mice show lower survival rates when infected with mouse cytomegalovirus (MCMV) compared with wild-type (WT) mice (33). HIV-1 RNA increases GCN2 kinase activity in cell-free extracts and transfected cells; however, HIV-1 infection of a human T cell line leads to cleavage of GCN2 by HIV-1 protease, showing that HIV-1 not only induces GCN2, but it also antagonizes its function (36). GCN2 silencing by siRNA increases HIV-1 infectivity, concomitant with an increase in new protein synthesis (37), consistent with GCN2 being antiviral through inhibition of translation. During viral infection, GCN2 may be activated by two main mechanisms: amino acid depletion with accumulation of uncharged tRNAs or ribosome collisions stimulated by ribosome stalling (34, 38). Infection of human dendritic cells by live attenuated yellow fever vaccine virus (YF-17D) leads to a reduction in arginine and an increased phosphorylation of GCN2 (39). Another flavivirus, Zika virus (ZIKV), also leads to GCN2 phosphorylation; in ZIKV infections, GCN2 activation is dependent on viperin-induced translation inhibition triggered by colliding ribosomes (40). GCN2 activation has a broad impact on cell survival and on immune responses during infection, regulating a fine-tuned balance between autophagy, apoptosis, and cell cycle progression (41–43).

Because MAV-1 infection leads to a reduction of the ISR PKR protein kinase levels by proteasomal degradation (24, 25), we hypothesized that GCN2 also plays a role in MAV-1 pathogenesis. We found that GCN2 is more highly phosphorylated in MAV-1-infected cells and GCN2 activation is dependent on viral replication. *Gcn2^−/^*^−^ mice infected with MAV-1 had lower survival than wild-type mice, indicating that GCN2 has an antiviral role during MAV-1 infection. However, infection of the *Gcn2^−/^*^−^ mice did not lead to higher viral loads in brain and spleen than WT mice. This suggests that the difference in survival between *Gcn2^−/^*^−^ and WT mice was not due to increased viral replication in *Gcn2^−/^*^−^ mice but rather a more complicated interplay between viral infection and the host response. Histopathologic findings of multifocal parenchymal microhemorrhages were seen in both *Gcn2^−/^*^−^ and WT mice, and they were more abundant in the *Gcn2^−/^*^−^ animals. However, the microhemorrhages alone could not explain the higher mortality of *Gcn2^−/^*^−^ mice. Therefore, we evaluated cytokine levels by both RNA and protein assays. Of all the cytokines analyzed, only IL-1β levels differed between the strains. Because GCN2 signaling can also regulate the balance between autophagy induction and inflammasome activation (41), we investigated the levels of IL-1β production *in vitro* and observed higher levels in *Gcn2^−/^*^−^ cells compared to WT. Our results also suggest that GCN2 (and not PKR) is the primary inducer of phosphorylated-eIF2α (peIF2α) during MAV-1 infection.

## RESULTS

### MAV-1 infection leads to GCN2 phosphorylation in cultured mouse embryo fibroblasts (MEFs)

**Fig 1:**
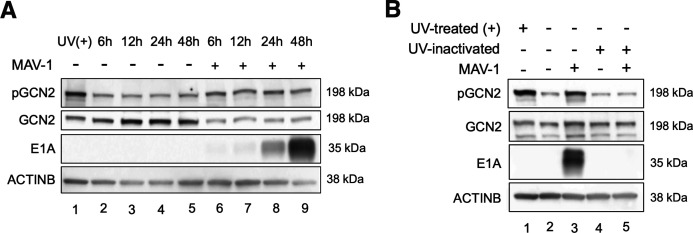
MAV-1 infection leads to GCN2 phosphorylation. (**A**) Wild-type (C57BL/6) MEFs were either treated with UV to induce GCN2 phosphorylation (UV (+), positive control, lane 1), mock infected with conditioned media (lanes 2–5), or infected with MAV-1 at an MOI of 5 (lanes 6–9) and collected at 6, 12, 24, and. 48 hpi. Cells were lysed with Laemmli buffer, and the designated proteins were evaluated by immunoblot. (**B**) WT (C57BL/6) MEFs were either treated with UV (UV (+), positive control, lane 1), mock-infected with conditioned media (lane 2) or UV-treated conditioned media (lane 4), infected with MAV-1 at an MOI of 5 (lane 3) or a corresponding amount of UV-inactivated MAV-1 (lane 5), and collected 24 hpi. Cells were lysed with Laemmli buffer, and the designated proteins were evaluated by immunoblot. Data are representative of two independent experiments.

To evaluate whether MAV-1 infection induces phosphorylation of GCN2, we infected C57BL/6J MEFs with MAV-1 at an MOI of 5 and harvested cell lysates at 6, 12, 24, and 48 hours post-infection (hpi). As a positive control for GCN2 activation by phosphorylation, we UV-treated the cells with 50,000 μJ/cm^2^ and allowed 30 minutes for recovery. We used a detection method to optimize visualizing phosphorylated GCN2 (pGCN2) and GCN2 levels that involves treatment with hydrogen peroxide after the first (pGCN2) antibody probing (instead of harsh stripping [44, 45]). Due to steric hindrance of the anti-pGCN2 antibody persisting after the hydrogen peroxide treatment, it is likely that only nonphosphorylated GCN2 will be detected by the anti-GCN2 antibody. We assayed pGCN2 levels by first immunoblotting with an antibody specific for pGCN2. The levels of pGCN2 were higher at 6 hpi compared to uninfected cells and remained elevated up to 48 hours (Fig. 1A). At the same time, the levels of nonphosphorylated GCN2, detected by an antibody that should recognize both pGCN2 and GCN2, were lower in infected cells compared to uninfected cells. We used E1A protein detection to confirm viral infection. Very low levels of E1A were detected at 6 hpi and increased as infection progressed, reaching a very high level at 48 hours (Fig. 1A). Using UV-inactivated virus, we also evaluated whether the virus particles could activate GCN2 or whether the virus needed to be actively replicating for GCN2 to become phosphorylated. We infected C57BL/6J MEFs with MAV-1 MOI of 5 or treated these cells with an equivalent amount of UV-inactivated virus (Fig. 1B). Only inoculation with the non-UV-treated MAV-1 resulted in phosphorylated GCN2 (lane 3); the levels of GCN2 phosphorylation were similar between cells treated with the UV-inactivated virus and uninfected cells (lanes 5 and 4, respectively). These results demonstrate that MAV-1 infection leads to GCN2 phosphorylation, and GCN2 phosphorylation is dependent on virus replication.

### Absence of GCN2 results in increased MAV-1 yield in cultured cells

**Fig 2:**
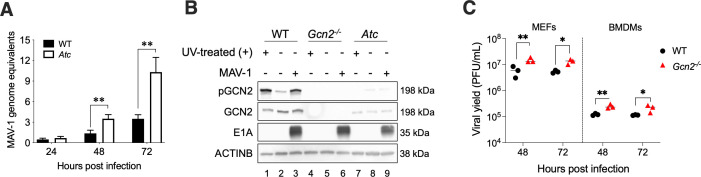
Absence of GCN2 results in increased MAV-1 yield in cultured cells. (**A**) WT (C57BL/6) and *Atc* (*GCN2^m1Btlr^*, *Atchoum*) peritoneal macrophages were infected with MAV-1 at MOI 1 and collected at 24, 48, and 72 hpi. DNA was extracted from cells and analyzed by qPCR for E1A fold-change; MAV-1 genome equivalents were normalized to 24 hpi. Graphs are representative of n = 15 biological replicates by group. Error bars are standard error of the mean (SEM). Asterisks indicate a statistically significant difference between *Atc* and WT mice by Mann-Whitney test (**P < 0.01). (**B**) WT (C57BL/6), *Gcn2^−/^*^−^ and *Atc* MEFs were either treated with UV to induce GCN2 phosphorylation (UV (+), positive control, lanes 1, 4, and 7), mock-infected with conditioned media (lanes 2, 5, and 8), or infected with MAV-1 at MOI 5 (lanes 3, 6, and 9) and collected at 24 hpi. Cells were lysed with Laemmli buffer, and the designated proteins were evaluated by immunoblot. (**C**) WT (C57BL/6) and *Gcn2^−/^*^−^ MEFs and BMDMs were infected with MAV-1 at MOI 5 and harvested at 48 and 72 hpi. Virus titers were determined by plaque assay in 3T6 cells. All infections were performed in biological triplicates at each time point. This result is representative of three independent experiments. Error bars represent the standard error of the mean (SEM). Asterisks indicate a statistically significant difference of *Gcn2^−/^*^−^ mice compared to WT mice by Mann-Whitney test (*, *P* < 0.05; **, *P* < 0.01).

To determine whether the GCN2 (*Atc*) mutation leads to an increased yield of virus following infection with MAV-1, we infected peritoneal macrophages isolated from *Atchoum (Atc*) mice and WT (C57BL/6J) mice. We infected cells at an MOI of 1 and harvested cells and supernatants at 24, 48, and 72 hpi for DNA extraction. MAV-1 DNA levels were evaluated by qPCR. No appreciable difference in DNA levels was detected between peritoneal macrophages of the two strains at 24 hpi (Fig. 2A). However, at both 48 and 72 hpi, the viral DNA yields from *Atc* macrophages were higher than those from WT macrophages. This suggests that GCN2 is antiviral during MAV-1 infection.

We evaluated GCN2 phosphorylation levels in *Atc* MEFs infected with MAV-1 at MOI of 5 (Fig. 2B, lanes 7–9). We observed a faint pGCN2 band in the immunoblots from cellular extracts (lane 8) and GCN2 bands in lanes 7–9. The *Atc* mutation corresponds to a thymine-to-cytosine transition of the sixth nucleotide of intron 2 of *Eif2ak4*, encoding GCN2, leading to exon 2 skipping in peritoneal macrophages, and in some cases skipping of exons 3 and 4 (33). This was reported to produce no GCN2 in peritoneal macrophages. However, in addition to the *Atc* MEF results here, we detected the expression of GCN2 protein from additional *Atc* cell types (data not shown). Mice with a different GCN2 mutation, *Eif2ak4^tm1.2Dron^* mice (46), referred to here as *Gcn2^−/^*^−^ mice, have a deletion of exon 12 of the *Eif2ak4* (*GCN2*) gene. MEFs from the *Gcn2^−/^*^−^ mice did not have GCN2 or pGCN2 protein bands (Fig. 2B, lanes 4–6). Because the mutation of *GCN2* appeared to be leaky in *Atc* cells, we continued our subsequent experiments with the *Eif2ak4^tm1.2Dron^* (*Gcn2^−/^*^−^) mice (46). In both MEFs and bone marrow-derived macrophages (BMDMs) isolated from *Gcn2^−/^*^−^ mice, MAV-1 replicated to higher levels than in WT cells both at 48 and 72 hpi, as determined by plaque assay (Fig. 2C). These results support the earlier data with *Atc* mice suggesting that GCN2 has an antiviral role during infection because the virus replicates to higher levels in cells lacking GCN2.

### Mice deficient in GCN2 production have lower survival than WT mice after sublethal MAV-1 challenge but no difference in viral load or blood-brain barrier disruption

**Fig 3:**
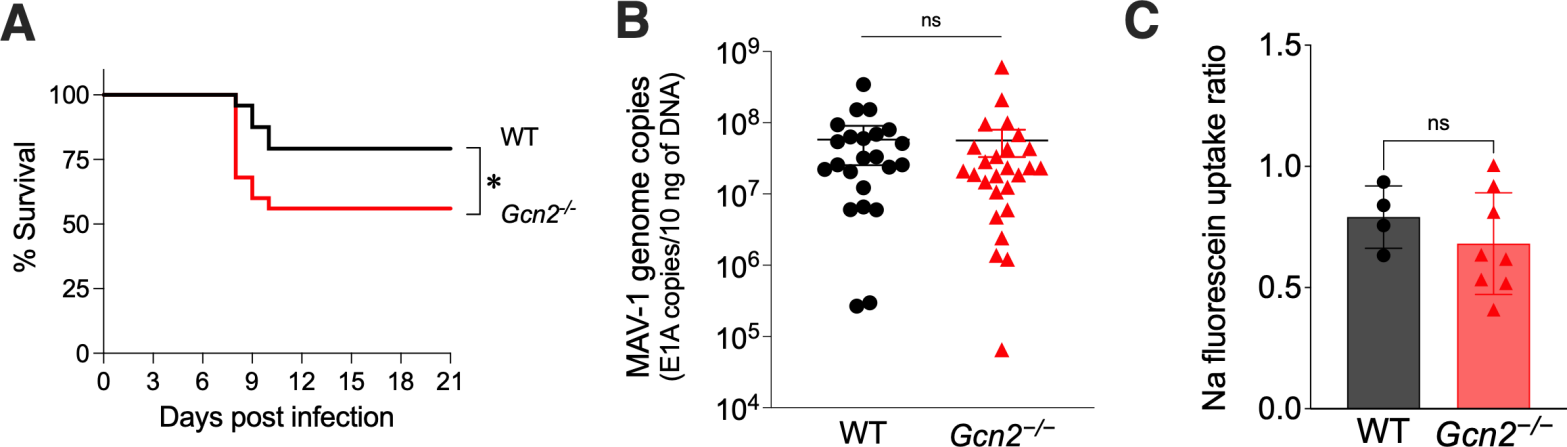
*Gcn2^-/^***^-^** mice are more susceptible to MAV-1 infection than WT mice. (**A**) Survival analysis after i.p. inoculation with 10^2^ PFU of MAV-1. WT (n = 24) mice are represented in black, and *Gcn2 ^−/^*^−^ (n = 25) mice are represented in red. The data shown are pooled from independent experiments. The asterisk indicates a statistically significant difference between *Gcn2 ^−/^*^−^ mice and WT mice by the Gehan-Breslow-Wilcoxon test (*, *P* < 0.05). (**B**) MAV-1 replication in brains from WT (C57BL/6J) and GCN2 *^−/^*^−^ mice 8 dpi. WT mice (n = 23) are represented by closed black circles, and GCN2*^−/^*^−^ mice (n = 26) are represented by closed red triangles. Viral loads were assessed by qPCR for viral DNA. The limit of detection of the assay was 10^4^ viral genome copies/10 ng of DNA. There was no statistically significant difference (ns) of *Gcn2^−/^*^−^ mice compared to WT mice by the Mann-Whitney test. (**C**) BBB permeability was assessed by sodium fluorescein uptake assay. WT (C57BL/6J) and *Gcn2 ^−/^*^−^ mice were injected i.p. with sodium fluorescein 10 min prior to euthanasia at 8 dpi. Uptake of sodium fluorescein (μg) in 1 g of brain was normalized to sodium fluorescein (μg) in the serum (μL). WT (C57BL/6J) (n = 4) mice are represented by black circles, and *Gcn2^−/^*^−^ (n = 8) mice are represented by red triangles. There was no statistically significant difference of *Gcn2^−/^*^−^ mice compared to WT mice by the Mann Whitney test.

The role of GCN2 during MAV1 viral encephalitis is unknown. To characterize the *in vivo* physiological role of GCN2 in protection from MAV-1 infection, we compared survival of WT mice and *Gcn2^−/^*^−^ mice after intraperitoneal (i.p.) infection with 10^2^ PFU of MAV-1. There was a statistically significant difference in survival: ~80% of WT mice survived compared to ~55% of the mutant mice (Fig. 3A). For subsequent experiments, we used a dose of 10^2^ PFU/mouse and assayed at 8 days post-infection (dpi) (when significant mortality was observed) to examine parameters that might differ between WT and *Gcn2^−/^*^−^mice.

The highest levels of MAV-1 in infections are found in brains and spleens (27, 47–49). We quantitated MAV-1 virus present in the brains and spleens of WT and *Gcn2^−/^*^−^ mice 8 dpi with 10^2^ PFU MAV1 by measuring viral DNA levels by qPCR. We did not observe any difference in viral DNA levels between WT and GCN2 mouse brains (Fig. 3B) or spleens (data not shown). Additionally, we evaluated infectious virus levels by plaque assay from randomly selected samples. There was a good correlation between plaque assay titers and MAV-1 genome copies measured by qPCR, and there was also no difference in virus titers between WT and *Gcn2^−/^*^−^ mice (data not shown). We also measured MAV-1 viral DNA levels in brains of *Atc* and WT mice at 3, 5, and 7 dpi and did not observe any differences between the strains (data not shown).

MAV-1 infection in mice leads to the disruption of the blood-brain barrier (BBB) (50). To determine whether the difference in mouse susceptibility seen in Fig. 3A correlated with any difference in BBB disruption, we assayed BBB permeability to sodium fluorescein. Sodium fluorescein is a small molecule (376 Da) that can only access and stain brain tissue when the BBB is compromised. We administered sodium fluorescein i.p. at 8 dpi to WT and *Gcn2^−/−^* mice, and we quantitated the sodium fluorescein present in the right brain hemispheres. There was no statistical difference in sodium fluorescein uptake in brains from WT and *Gcn2^−/−^* mice (Fig. 3C). Although GCN2 plays a role in the survival of mice infected with MAV-1, it does not affect the viral replication levels nor the blood-brain barrier disruption in these mice.

### MAV-1 pathogenesis in WT and *Gcn2^−/^*^−^ mice

**Fig 4:**
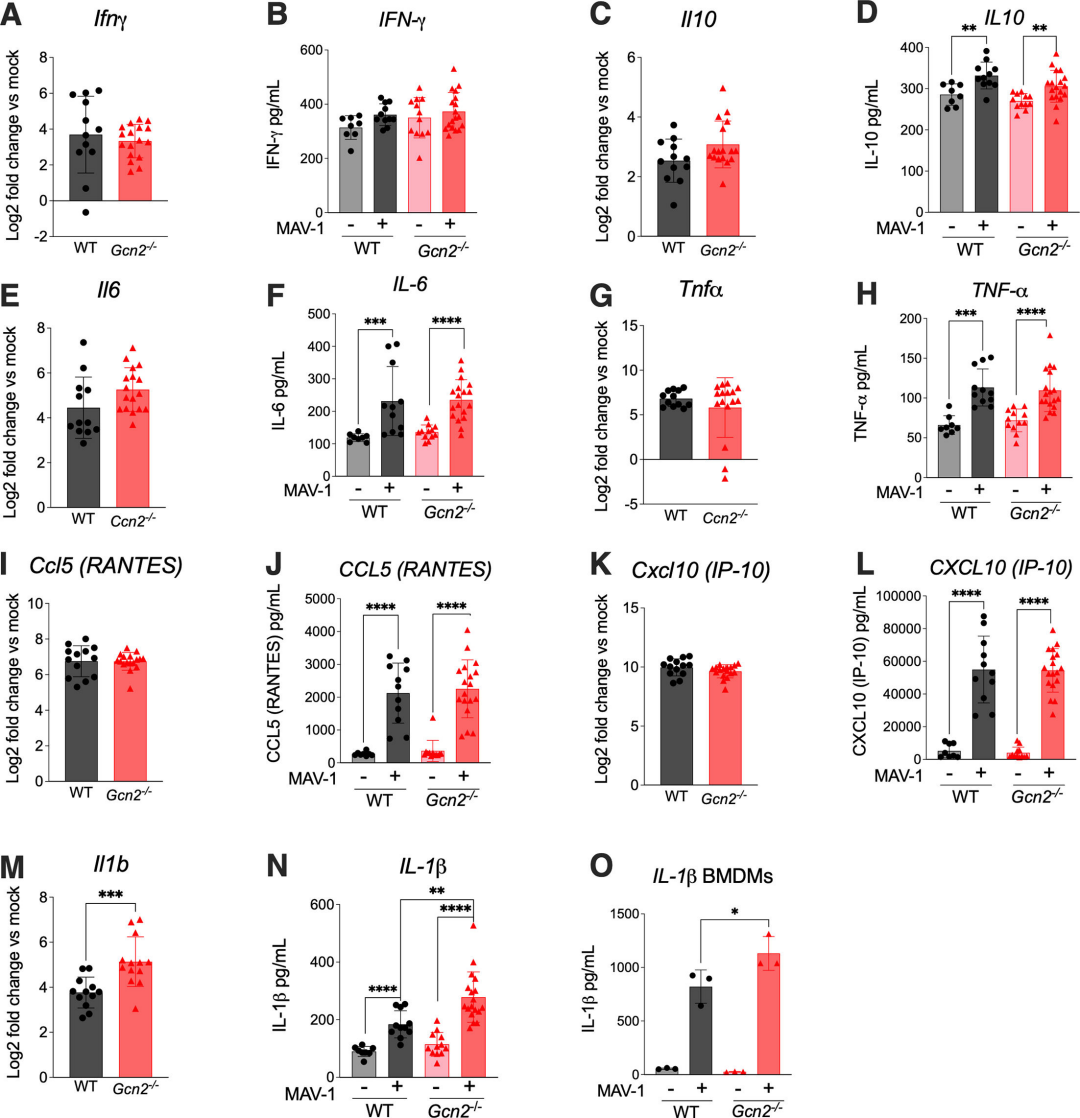
Only IL-1β levels are higher in brains of *Gcn2^−/^*^−^ mice compared to WT during MAV-1 infection. WT (C57BL6/J) and *Gcn2^−/^*^−^ mice were infected with 10^2^ PFU of MAV-1 or conditioned media. Brains were harvested at 8 dpi, and the RNA was extracted for qPCR analysis (**A, C, E, G, I, K, M**) and protein was extracted for quantification by ELISA (**B, D, F, H, J, L, N, O**). (**O**) WT (C57BL/6) and *Gcn2^−/^*^−^ BMDMs were primed with 0.2 µg/mL LPS, infected with MAV-1 at MOI 10, and harvested at 24 hpi. Statistical analysis was performed by Mann-Whitney test. *, *P* < 0.05; **, *P* < 0.01; ***, *P* < 0.001; ****, *P* < 0.0001.

One possible explanation for the higher mortality in *Gcn2^−/^*^−^ mice compared to WT mice without accompanying higher viral load or higher BBB disruption is that the absence of GCN2 could lead to an altered immune response that contributes to the disease severity. We analyzed the levels of chemokines and cytokines in brains of WT and *Gcn2^−/^*^−^ mice infected with 10^2^ PFU of MAV-1 and harvested at 8 dpi. We evaluated RNA levels by qPCR and protein levels by ELISA. We observed a large difference between the levels of IL-6, TNFα, IL-10, CCL5, and CXCL10 in mock and infected mice for both strains; however, there was no difference between the strains (Fig. 4A through L). In contrast, IL-1β levels were higher in *Gcn2^−/^*^−^ mouse brains compared to WT mouse brains, as measured by both qPCR and ELISA (Fig. 4M and N). Because GCN2 signaling can regulate the balance between inflammasome activation and autophagy induction (41), we investigated IL-1β production in BMDMs isolated from WT and *Gcn2^−/^*^−^ mice and observed higher levels of IL-1β in *Gcn2^−/^*^−^ BMDMs (Fig. 4O).

**Fig 5:**
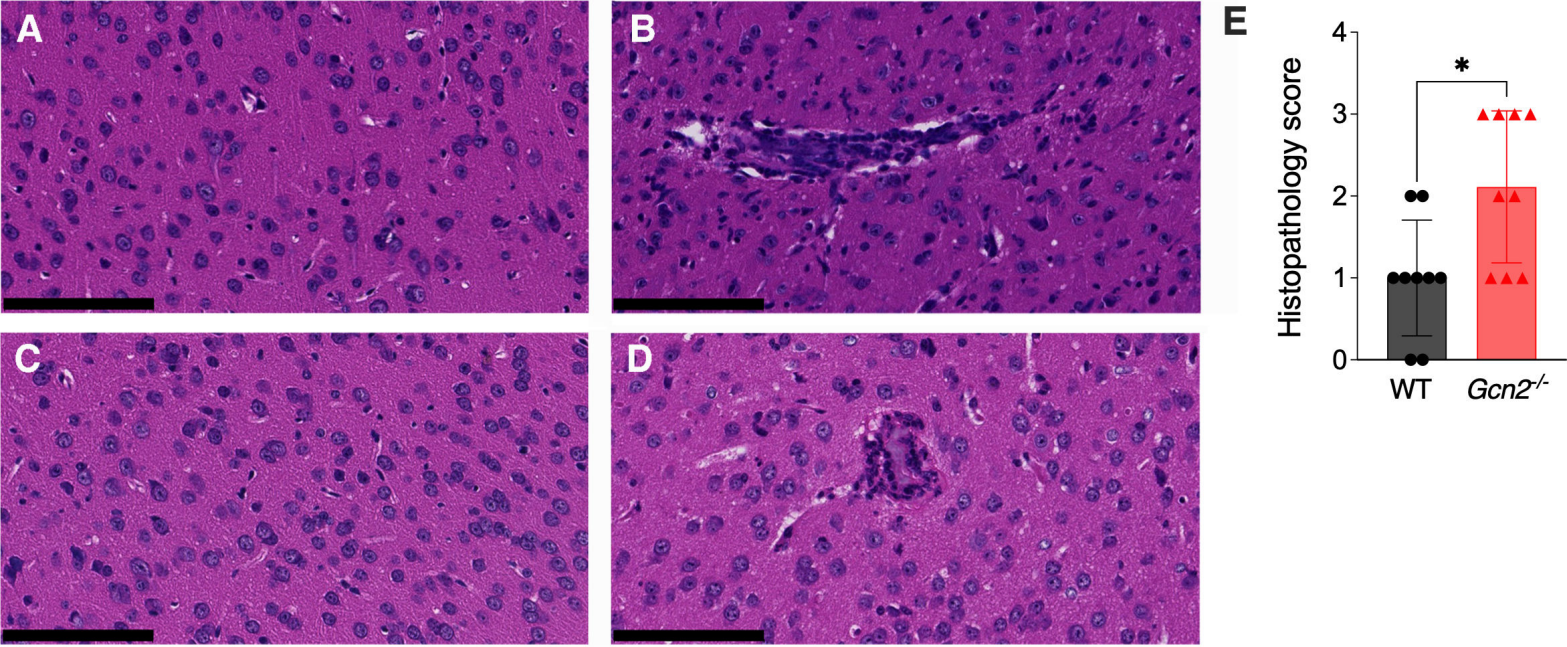
Histopathology of WT and*Gcn2^−/^*^−^ mice. C57BL/6 (**A, B**) and *Gcn2^−/^*^−^ (**C, D**) mice were mock-infected or infected with 10^2^ PFU MAV-1 i.p. Brain sections were prepared 8 dpi from mock-infected mice (**A, C**) or infected mice (**B, D**). Sections were stained with hematoxylin and eosin. Scale bar, 100 µm. (**E**) Histological scores of brain damage for WT and *Gcn2^−/^*^−^ mice. Data points represent individual mice, and the bars indicate arithmetic mean values. Statistical analysis was performed by Mann-Whitney test. *, *P* < 0.05.

To better understand the difference in mouse survival and the higher IL-1β present in the brains of *Gcn2^−/^*^−^ mice, we evaluated the histopathological findings in WT and *Gcn2^−/^*^−^ mice infected with 10^2^ MAV-1 PFU. Microscopic findings at 8 dpi in the brains of MAV-1-infected mice consisted of multifocal encephalitis and meningitis with vasculitis and perivascular edema (Fig. 5A through D). Lesions contained infiltrates of neutrophils with few macrophages. The severity and distribution of the lesions were quantified by blinded scoring of histological sections of brains (Fig. 5E). Although *Gcn2^−/^*^−^ mice had worse histopathological scores than WT mice, the overall pathology in both strains was mild, suggesting mortality was unlikely due to these lesions alone. No differences were seen between the strains for other organs examined (thymus, lung, heart, brain, liver, kidney, and spleen). While both strains showed mild histopathological changes and similar cytokine profiles for most markers, the distinct increase in IL-1β in *Gcn2^−/^*^−^ mice suggests a unique inflammatory response contributing to their increased disease severity.

### eIF2α phosphorylation during MAV-1 infection is predominantly due to GCN2 activation rather than PKR activation

**Fig 6:**
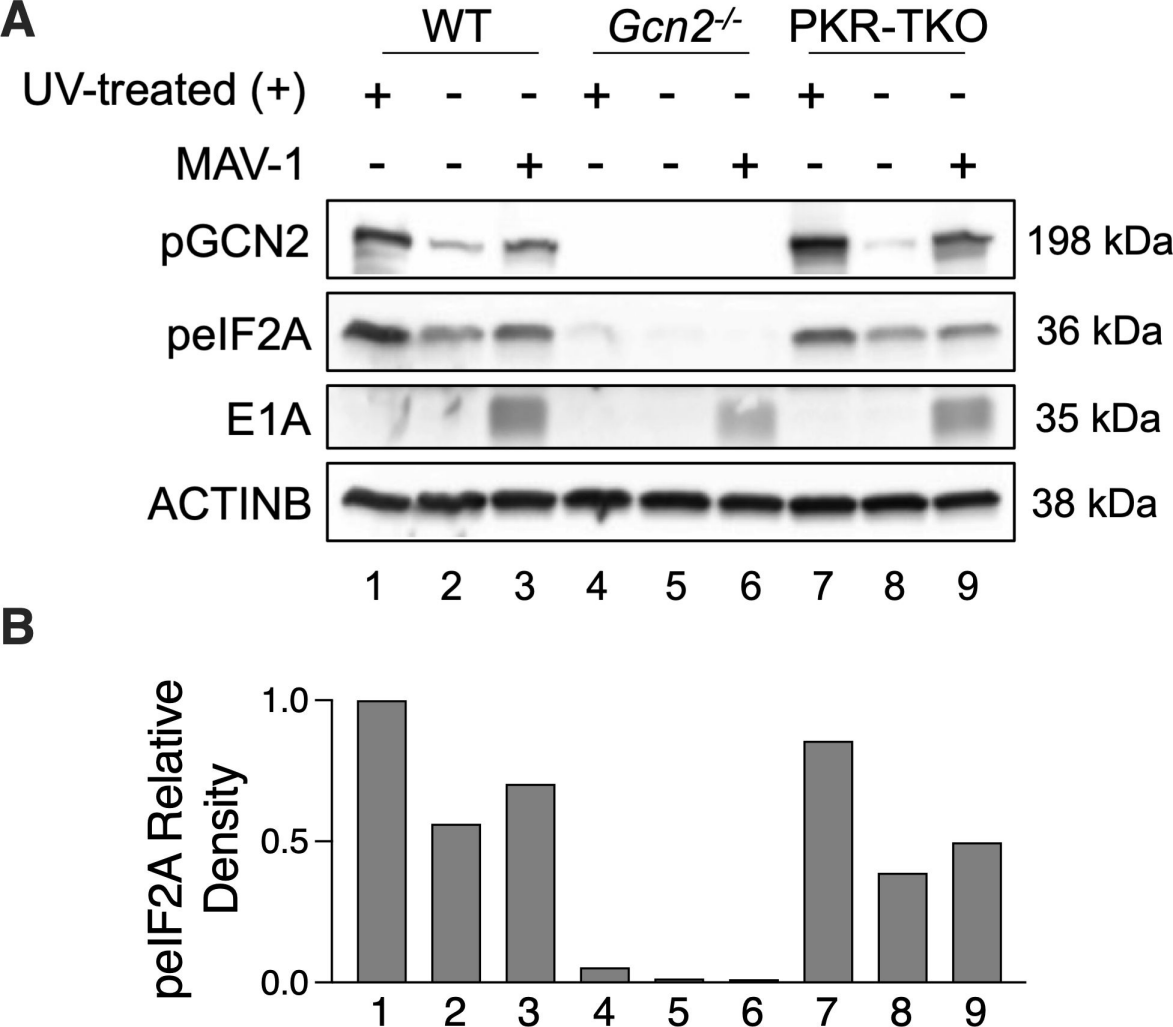
eIF2α phosphorylation during MAV-1 infection is mainly due to GCN2 activation. (**A**) WT (C57BL/6), *Gcn2^−/^*^−^ and PKR-TKO MEFs were either treated with UV to induce GCN2 phosphorylation (UV (+), positive control, lanes 1, 4, and 7), mock-infected with conditioned media (lanes 2, 5, and 8), or infected with MAV-1 at an MOI of 5 (lanes 3, 6, and 9) and collected at 24 hours post-infection. Cells were lysed with Laemmli buffer, and the designated proteins were evaluated by immunoblot. The experiment was performed three times, and a representative image is shown. (**B**) The density for peIF2A was calculated by the percent of area density relative to the WT UV positive control (Lane 1) and then normalized to the relative density of ACTINB.

To examine whether eIF2α is responsible for the increased susceptibility of *GCN2^−/^*^−^ mice to MAV-1 infection when compared to WT, we used cells that do not express PKR, PKR-TKO MEFs (32). We evaluated eIF2α phosphorylation levels in WT, *Gcn2^−/^*^−^ and PKR-TKO MEFs infected with MAV-1 at an MOI of 5 (Fig. 6A) and found little to no eIF2α phosphorylation in cells lacking GCN2. We observed only a faint peIF2α band in immunoblots from UV-stimulated positive control *Gcn2^−/^*^−^ cellular extracts (lane 4) and almost no band in MAV-1-infected *Gcn2^−/^*^−^ MEFs (lane 6), confirmed by peIF2α densitometry analysis (Fig. 6B). In contrast, WT and PKR-TKO MEFs exhibited peIF2α bands in positive controls (lanes 1 and 7) and MAV-1-infected MEFs (lanes 3 and 9) (Fig. 6), with higher peIF2α levels in infected cells compared to mock. We have shown that MAV-1 very effectively degrades PKR, such that there is almost no PKR detectable during infection (24). However, eIF2α phosphorylation still occurred at high levels in the absence of PKR, but not in the absence of GCN2 (Fig. 6). Because the lack of GCN2 nearly eliminated eIF2α phosphorylation in MAV-1-infected MEFs, whereas the lack of PKR did not alter eIF2α phosphorylation levels, these data suggest that GCN2 is more responsible for eIF2α phosphorylation than PKR during MAV-1 infection.

## DISCUSSION

We sought to determine the role of GCN2 during MAV-1 infection. We evaluated whether MAV-1 activates GCN2 and the effects of this antiviral pathway during infection. We demonstrated that MAV-1 induces the phosphorylation of GCN2 (Fig. 1A and B), which has been implicated in the pathogenesis of many viruses (33–36). GCN2 is antiviral in MAV-1 infections of cultured cells, and lack of GCN2 leads to a decrease in mouse survival during MAV-1 infection (Fig. 2 and 3). This higher mortality was not due to higher viral replication or an increase of BBB disruption in infected mouse brains. Instead, we showed that GCN2 deficiency leads to higher IL-1β levels in infected mouse brains (Fig. 4), suggesting that there could be an imbalance of autophagy and inflammasome activation upon virus infection, as described for other virus infections (39). We also showed that GCN2 is the primary inducer of eIF2α phosphorylation during MAV-1 infection and that eIF2α phosphorylation occurs even in the absence of PKR (Fig. 6).

GCN2 phosphorylation can be induced by amino acid starvation and a decrease in uncharged tRNAs or by ribosome stalling (38, 40). We have shown that during MAV-1 infection, GCN2 is phosphorylated, and viral replication is required for its activation. Exposing MAV-1 to UV-irradiation renders it biologically inactive (confirmed by plaque assay of UV-treated MAV-1 and the absence of E1A gene expression in Fig. 1B, lane 5). UV-inactivated virus was unable to induce phosphorylation of GCN2 (Fig. 1B). The same is observed for yellow fever virus in human dendritic cells, in which only actively replicating virus causes a depletion in amino acid pools, specifically in arginine levels (39). Importantly, we showed that GCN2 signaling has an antiviral role during MAV-1 infection. *Gcn2^−/^*^−^-infected MEFs and BMDMs produced more infectious virus than did WT cells (Fig. 2C). This correlates with the fact that GCN2 inhibits virus replication of HIV-1 and VSV (37, 51). In addition, overexpression of GCN2 in a MEF cell line severely reduced Sindbis virus (SINV) replication (35).

When investigating the impact of GCN2 activation during MAV-1 infection *in vivo*, we observed that GCN2 also played a protective role in mice. There was an increase in mortality of almost 30% after MAV-1 infection in the absence of GCN2. Similarly, mice infected with MCMV show around 20% higher mortality when they lack functional GCN2, compared to mice with functional GCN2 (33). However, there were no differences in MAV-1 viral replication in the brains or spleens of WT and *Gcn2^−/^*^−^ mice. While other organs were not examined for viral loads, we did not see differences in histopathology in organs other than the brain. During SINV infection, GCN2 is important for viral defense during the early stages of infection (35). At 3–4 dpi, *Gcn2^−/^*^−^ mice have significantly higher SINV titers in their brains compared to WT animals, while at 5 dpi, there were no differences in viral titers in infected *Gcn2^−/^*^−^ and wild-type mouse brains (35). We measured MAV-1 viral loads in *Atc* and wild-type mice brains at 3- and 5-days post infection and did not observe any differences between the strains (data not shown).

We hypothesized that the lack of GCN2 could elicit a different inflammatory response after MAV-1 infection, leading to the higher mortality rate observed in *Gcn2^−/^*^−^ mice. Examination of histopathology induced by MAV-1 infection only revealed differences in brains and not other organs. Brains from both WT and *Gcn2^−/^*^−^ mice indicated the presence of multifocal encephalitis and meningitis with vasculitis and perivascular edema. However, even though *GCN2^−/^*^−^ mice had worse histopathological scores than WT mice overall, the lesions were generally mild, and therefore mortality seems unlikely to be due to these lesions alone. We also evaluated the brain uptake of sodium fluorescein after MAV-1 infection in WT and *Gcn2^−/^*^−^ mice since it can be used to detect BBB disruption and presence of microhemorrhages. We did not observe any difference in brain sodium fluorescein uptake between WT and *Gcn2^−/^*^−^ mice infected with MAV-1 (Fig. 3C). This contrasts with our observation of slightly more brain pathology in the *Gcn2^−/^*^−^ mice. However, a caveat is that we evaluated global fluorescein uptake by homogenizing the whole brain and assaying fluorescence. Detecting sodium fluorescein uptake by imaging would be more sensitive to detect small, localized changes since there can be a variation of uptake levels depending on the brain area (52–54).

GCN2 is involved in the development of the remission phase of experimental autoimmune encephalomyelitis in C57BL/6 (WT) mice (55). When WT and GCN2 KO mice were immunized with myelin oligodendrocyte glycoprotein peptide, GCN2 KO mice did not develop the remission phase of the disease, and this was associated with higher levels of CNS inflammation and increased presence of effector T cells (Th1/Th17). When evaluating histological sections of the lumbar spinal cord, the presence of the inflammatory cells was more evident in GCN2 KO mice, especially at the remission phase of the disease at day 21, compared to the peak phase at day 15 post-immunization. Upon examination of the cytokines involved in MAV-1 pathogenesis, both by qPCR and ELISA in mouse brains, we only noted altered levels of one: we detected elevated levels of only IL-1β in brains of *Gcn2^−/^*^−^ mice compared to WT mice after MAV-1 infection. The small difference in the cytokines and histopathological alterations in these mice indicate that there are other factors affecting mouse survival that should be explored in this system.

GCN2 is critical to the maintenance of cellular and organismal homeostasis and plays multiple roles in immune cell functions during viral infection (34, 43). Virus-induced GCN2 activation has a key role in programming dendritic cells to initiate autophagy and enhance antigen presentation to both CD4 and CD8 T cells in the context of yellow fever 17D vaccine (39). The yeast eIF2α kinase GCN2 and the eIF2α-regulated transcriptional transactivator GCN4 are essential for starvation-induced autophagy, and notably, PKR can rescue GCN2-disrupted yeast from starvation-induced autophagy (56). Divergent stress stimuli such as nutrient deprivation and herpes simplex virus infection stimulate eIF2α kinase-dependent translational arrest, and they also stimulate eIF2α kinase-dependent autophagy. GCN2 is also involved in controlling intestinal inflammation by suppressing inflammasome activation in a mouse model of acute colitis (41). Genetic deletion of GCN2 in CD11c^+^ antigen-presenting cells or intestinal epithelial cells results in enhanced intestinal inflammation and T helper 17 cell (TH17) responses and a reduction in autophagy, leading to increased reactive oxygen species (ROS), with enhanced inflammasome activation and IL-1β production (41). During MAV-1 infection, GCN2 may help balance inflammasome activation and autophagy since IL-1β levels were higher by both qPCR and ELISA in *Gcn2^−/^*^−^ mouse brains compared to WT mouse brains (Fig. 4M and N). Further investigation is necessary to further determine whether the higher mortality observed in the absence of GCN2 is connected to a decrease in autophagy and increase in inflammasome activation.

We determined that the presence of GCN2 in MEFs was required to increase eIF2α phosphorylation during MAV-1 infection (Fig. 6, lanes 5 and 6). In contrast, the presence of PKR did not affect eIF2α phosphorylation (Fig. 6, lanes 8 and 9). This indicates that of these two eIF2α-kinases, GCN2 is the primary one phosphorylating eIF2α during MAV-1 infection. Similar findings are observed when MEFs are infected with murine norovirus, and virus-induced eIF2α phosphorylation is impaired in *GCN2^−/^*^−^ MEFs compared to WT (57). The extent of eIF2α phosphorylation will influence the degree to which global protein synthesis is reduced (58). Strong eIF2α phosphorylation can halt global translation, while weak eIF2α phosphorylation may not affect all translation. Considering the effects of a viral infection on cellular homeostasis from the cells’ standpoint, the impairment of protein synthesis would be desired to prevent or reduce viral replication. We hypothesize that during MAV-1 infection, GCN2 is the primary eIF2α-kinase phosphorylating eIF2α, while PKR does not affect eIF2α phosphorylation levels. We have demonstrated that there is substantial viral degradation of PKR induced by MAV-1 infection (24), and we show here that a lack of PKR does not affect total eIF2α phosphorylation. This indicates that other functions of PKR are important during infection (32) and that GCN2 is the main eIF2α-kinase involved in ISR activation in the context of MAV-1 infection. It will be necessary to study this mechanism further to determine whether GCN2 activation leads to an alteration in the balance between inflammasome and autophagy.

## MATERIALS AND METHODS

### Virus and cells

MAV-1 originally obtained from S. Larsen (59) was grown and titrated in 3T6 fibroblasts, as described previously (60). Conditioned media used for mock infections was prepared from uninfected 3T6 cells in a similar manner. MAV-1 and conditioned media were UV-inactivated, as described (24); non-infectivity of virus was confirmed by plaque assay.

The mouse 3T6 cell line (ATCC CCL-96) was originally obtained from Dr. Alvin Winters, University of Alabama, Tuscaloosa, and cells were maintained in Dulbecco’s modified Eagle’s media (DMEM) containing 5% heat-inactivated newborn bovine serum (HINS). *Atc* MEFs and *Gcn2^−/^*^−^ MEFs were derived from Atc and *Gcn2^−/^*^−^ 15 day mouse embryos, as described previously (25), and PKR-TKO MEFs were derived from PKR-TKO mice (32). WT C57BL/6 mice used for infection and MEF production were housed in the same animal breeding room as mutant strains. *Atc*, PKR-TKO, and WT MEFS were maintained in DMEM containing 10% fetal bovine serum (FBS), and *Gcn2^−/^*^−^ MEFs were maintained in DMEM containing 20% fetal bovine serum, 1% non-essential amino acids (NEAA), and 1% MEM amino acids (GIBCO 11140-050 and 11130-051, respectively).

Primary peritoneal macrophages were isolated from 6-to-10-week-old C57BL/6J mice, as described previously (61). Primary BMDMs were prepared by flushing DMEM into mouse tibia and femurs of male or female mice aged between 8 and 12 weeks. Cells were differentiated by incubation with DMEM supplemented with 2 mM L-glutamine, 1 mM sodium pyruvate, 30% L929 cell-conditioned medium, 20% heat-inactivated FBS, 1 mM penicillin-streptomycin (GIBCO 15140-122), and 2 mM non-essential amino acids (NEAA, GIBCO 11140-050). After 7 days in culture, BMDMs were harvested and seeded at the required density for each experiment. L-929 cells were cultured in DMEM supplemented with 2 mM L-glutamine, 1 mM sodium pyruvate, 1 mM NEAA, 10 mM HEPES, and 10% heat-inactivated FBS. All cells were incubated at 37°C in 5% CO_2_.

### Mice

Wild-type C57BL/6J (cat. no. 000664) and *B6.129S6-Eif2ak4^tm1.2Dron^/J* (cat. no. 008240, *Gcn2^−/^*^−^) were purchased from Jackson Laboratory (Bar Harbor, ME). The *Gcn2^−/^*^−^ mice have a deletion of exon 12 of the *Eif2ak4* (GCN2) gene (46). *Eif2ak4^m1Btlr^* (*Atc*) mice on the C57BL/6 background obtained from Bruce Beutler (UT Southwestern Medical Center) have an ENU-induced T to C transition at position 12,038 (GenBank NC_000068); the mutation affects the donor splice site of intron 2. The *Atc*, *Gcn2^−/^*^−^, and C57BL/6J mice were bred in-house, and both sexes were used in experiments. No differences based on sex in any assayed phenotypes were noted. *Atc* mutations were confirmed by genotyping mice as described (33). *Gcn2^−/^*^−^ mutations were confirmed by Transnetyx (Memphis, TN) based on the reported genotype (46). All animals were housed in specific pathogen-free facilities at the University of Michigan Medical School Unit for Laboratory Animal Medicine (ULAM). Animals were housed in microisolator cages and provided food and water *ad libitum*, and health checks were performed daily. Male and female mice were infected i.p. with the indicated virus dose diluted in endotoxin-free PBS in 0.1 mL, between the ages of 4 to 5 weeks. Mock-infected mice were infected with conditioned media prepared in parallel with the virus stock by collection of media from uninfected cells. Infected mice were housed in biosafety level 2 containment and treated in accordance with an IACUC-approved protocol. All animal work complied with relevant federal and University of Michigan policies.

### Quantitation of virus titers

MAV-1 titers in cells were determined either by qPCR or by plaque assay. When comparing *Atc* and WT peritoneal macrophages, cells were infected with MAV-1 at an MOI of 1. After 24, 48, and 72 hpi, cells were washed twice with room temperature PBS and harvested by scraping into PBS, centrifuging at 100  ×  *g* for 4 min at 4°C, and resuspending in PBS. Total cellular DNA was purified using an Invitrogen PureLink DNA purification kit (Thermo Scientific catalog no. K1820-02) and quantitated using a NanoDrop spectrophotometer. Total cellular DNA (10 ng) was analyzed by qPCR using custom primers specific to MAV-1 E1A (mE1Agenomic Fwd [5′ GCA CTC CAT GGC AGG ATT CT 3′] and mE1Agenomic Rev [5′ GGT CGA AGC AGA CGG TTC TTC 3′]), and the results were normalized to glyceraldehyde-3-phosphate dehydrogenase (GAPDH), which was analyzed using a GAPDH-specific primer/probe set (Thermo Fisher Scientific Mm99999915_g1; catalog no. 4331182). When comparing *Gcn2^−/^*^−^ and WT MEFs and BMDMs, cells were infected with MAV-1 at an MOI of 5. At 48 and 72 hpi, cells and supernatants went through three freeze-thaw cycles, were clarified by centrifugation at 500 × *g* for 10 min, and were stored at −80°C until titration in 3T6 cells by plaque assay (60).

MAV-1 viral DNA loads in brains and spleens were determined by qPCR with MAV-1 E1A genomic primers (62). Organs were harvested at 8 dpi, frozen, and DNA was extracted using the Invitrogen PureLink Genomic DNA Kit (K182002). DNA (10 ng) was analyzed by qPCR in 10 µL reactions. Real-time PCR was performed on an ABI Prism 7500 Fast Real-Time PCR System (Applied Biosystems), and the results were compared to a standard curve of plasmid containing known amounts of an E1A gene-containing plasmid to convert cycle threshold values to E1A DNA copy numbers. Each sample was assayed in triplicate.

### Antibodies

Blots for pGCN2 detection were first probed with the rabbit monoclonal Anti-GCN2 (phospho T899), which does not bind to nonphosphorylated GCN2 (Abcam, ab75836). To avoid removing GCN protein from the membrane by harsh stripping, the blots were briefly washed with 95% ethanol and then treated with 15% hydrogen peroxide for 30 min, as described (44). Then, to detect total GCN2, we used the rabbit monoclonal Anti-GCN2 (Cell Signaling, 3302). Before other primary antibody incubations, the ethanol/hydrogen peroxide treatment was repeated. To detect PKR, we used mouse monoclonal anti-PKR B-10 (Santa Cruz Biotechnology, sc-6282). To detect eIF2α, we used the rabbit polyclonal Anti-eIF2α (Invitrogen, AHO1182), and to detect phosphorylated eIF2α, we used the rabbit polyclonal Anti-eIF2α [pS52] (Invitrogen, 44728G). The primary antibody to MAV-1 E1A was 10B10, a mouse monoclonal described previously (63). Secondary antibodies for immunoblot were goat anti-rabbit-HRP and goat anti-mouse HRP (Kindle Biosciences R1006, and 1005, respectively). Before using the actin antibody, a harsh stripping method was used (rather than ethanol/hydrogen peroxide), which was a 15 min treatment at 56°C with 10% SDS, 62.5 mM Tris pH 6.8, and 0.8% 2-mercaptoethanol. We used a mouse monoclonal antibody to β-actin, (Santa Cruz Biotechnology, sc-47778) as a loading control, following all other antibody treatments.

### Preparation of cell lysates for immunoblot

Cell lysates for analysis of GCN2 were prepared as described (44), with minor modifications. Briefly, MEFs and BMDMs were plated in 12-well plates, with 4 × 10^5^ cells per well for MEFS and 1 × 10^6^ cells per well for BMDMs. Cells were infected with MAV-1 MOI of 5, and the virus inoculum was not removed. When ready to harvest, cells were washed once with DPBS, and then 80 µL of denaturing protein sample buffer (0.625M Tris-HCl pH 6.8; 10% glycerol; 3% SDS; 0.5 mM EDTA, 5% (vol/vol) 2-mercaptoetanol, 0.1% (wt/vol) bromophenol blue sample buffer) was added dropwise evenly across each well. The lysates were scraped and collected in 1.5 mL tubes and immediately snap-frozen. Before loading on gels, samples were incubated in a Thermomixer for 10 min at 99°C with 1,400 rpm of agitation.

### Immunoblot analysis

Lysates for immunoblots were prepared as described above and were analyzed by sodium dodecyl sulfate-polyacrylamide gel electrophoresis (SDS-PAGE) on 4%–15% gradient gels (BioRad 4561085). Gels were immunoblotted and blots visualized as described (25). Protein standards (Bio-Rad 1610374) were included in the gel as a size marker. ImageJ analysis for densitometry was performed using the Gel Analysis method (64).

### BBB permeability assay

Male and female WT and *Gcn2^−/^*^−^ mice, 4–5 weeks old, were injected i.p. with 10^2^ MAV-1. After 8 days of infection, 100 µL of 10% sodium fluorescein (Sigma) diluted in DPBS was injected i.p. 10 minutes prior to euthanasia. Cardiac blood was collected, and mice were transcardially perfused with 30 mL of DPBS. Brains were snap-frozen until used for quantitation. Sodium fluorescein levels in brain and serum were determined as previously described using the right brain hemisphere (65). Fluorescence levels were measured on a Bio-Tek microplate reader with a 485 nm excitation and a 530 nm emission. Sodium fluorescein standards were prepared in DPBS and used to calculate the sodium fluorescein content of brain and serum samples. Brain values were normalized to their respective serum dye values to allow comparisons among mice.

### Cytokine quantitation by qPCR and ELISA

For *in vivo* cytokine determination, WT and *Gcn2^−/^*^−^ mice, 4 to 5 weeks old, were infected with 10^2^ PFU of MAV-1, and brains were collected at 8 dpi. Approximately 50 mg of each brain was homogenized using sterile glass beads in a Mini-Beadbeater (Biospec Products) for 30 s in 1 mL of TRIzol (Invitrogen). RNA was then isolated from the homogenates according to the manufacturer’s protocol and stored at −70°C until use. Then, 1 µg of RNA was reverse-transcribed using the High-Capacity cDNA reverse transcription kit (Thermo Fisher Scientific) according to the manufacturer’s instructions. cDNA corresponding to 35 ng of RNA equivalent was used in each qPCR, and each sample was analyzed in triplicate. Quantitation was performed by normalizing target gene mRNA levels to β-actin levels, and infected sample values were expressed relative to the mean of mock values, set to 1 for each gene. To calculate the statistical significance of between-group differences, we used ΔCT and Log (2−ΔΔCT) values. Primers and probes used were as described (65).

For protein quantitation by ELISA in mouse brains, approximately 80 mg of each brain was processed as described (65). For *in vitro* cytokine determination, BMDMs were seeded overnight at a density of 2 × 10^5^ cells/well in 48-well plates and prestimulated with 0.2 µg/mL of bacterial lipopolysaccharide (LPS, Sigma-Aldrich L2630) for 4 h and subsequently infected with conditioned media or MAV-1 MOI of 10. The levels of cytokines and chemokines in mouse brains and cell culture supernatants were measured by ELISA at the University of Michigan Cancer Center Immunology Core. We measured IFN-γ, TNF-α, IL-1β, IL-6, IL-10, IP-10 (CXCL10), and RANTES (CCL5) proteins. Samples were stored at −70°C until use. Before use, samples were thawed on ice and centrifuged at 20,000 × *g* for 5 min at 4°C. Cytokine measurement (R&D and Peprotech) and protein quantitation by Pierce BCA protein assay kit (Thermo Scientific) were performed according to the manufacturer’s instructions.

### Histological analysis

WT and *Gcn2^−/^*^−^ mice were either mock-infected or infected with 10^2^ PFU of MAV-1, and at 8 dpi, organs were processed for histology. After euthanasia, mice were perfused with 30 mL 10% formalin (3.7% formaldehyde in PBS), and organs (thymus, lung, heart, brain, liver, kidney, and spleen) were immersion fixed in 10% neutral buffered formalin at 4°C. After 24 hours, organs were transferred to 70% ethanol and embedded in paraffin and sectioned at 4 µm. Sections were stained with hematoxylin and eosin. The University of Michigan Comprehensive Cancer Center Research Histology and Immunoperoxidase Laboratory performed sectioning and staining. Slides were randomized and blinded for evaluation by a board-certified pathologist. The sections were evaluated and scored as described (65).

### Data analysis

Statistical analyses were performed using Prism 10 (GraphPad Software, Inc.). Log-transformed values for viral load data were used for statistical comparisons. Differences between two groups were analyzed using the Mann–Whitney rank sum test. Comparisons made between groups at multiple time points were analyzed using two-way analysis of variance (ANOVA), followed by Sidak’s multiple-comparison tests. P values of < 0.05 were considered statistically significant.
